# Transcutaneous Auricular Vagus Nerve Stimulation Improves Spatial Working Memory in Healthy Young Adults

**DOI:** 10.3389/fnins.2021.790793

**Published:** 2021-12-23

**Authors:** Jin-Bo Sun, Chen Cheng, Qian-Qian Tian, Hang Yuan, Xue-Juan Yang, Hui Deng, Xiao-Yu Guo, Ya-Peng Cui, Meng-Kai Zhang, Zi-Xin Yin, Cong Wang, Wei Qin

**Affiliations:** ^1^Engineering Research Center of Molecular and Neuro Imaging of the Ministry of Education, School of Life Sciences and Technology, Xidian University, Xi’an, China; ^2^Intelligent Non-invasive Neuromodulation Technology and Transformation Joint Laboratory, Xidian University, Xi’an, China

**Keywords:** taVNS, working memory, n-back task, cognitive enhancement, non-invasive neuromodulation

## Abstract

Working memory (WM) is one of the core components of higher cognitive functions. There exists debate regarding the extent to which current techniques can enhance human WM capacity. Here, we examined the WM modulation effects of a previously less studied technique, transcutaneous auricular vagus nerve stimulation (taVNS). In experiment 1, a within-subject study, we aimed to investigate whether and which stimulation protocols of taVNS can modulate spatial WM performance in healthy adults. Forty-eight participants performed baseline spatial n-back tasks (1, 3-back) and then received online taVNS, offline taVNS, or sham stimulation before or during (online group) the posttest of spatial n-back tasks in random order. Results showed that offline taVNS could significantly increase hits in spatial 3-back task, whereas no effect was found in online taVNS or sham group. No significant taVNS effects were found on correct rejections or reaction time of accurate trials (aRT) in both online and offline protocols. To replicate the results found in experiment 1 and further investigate the generalization effect of offline taVNS, we carried out experiment 2. Sixty participants were recruited and received offline taVNS or offline earlobe stimulation in random order between baseline and posttests of behavioral tests (spatial/digit 3-back tasks). Results replicated the findings; offline taVNS could improve hits but not correct rejections or aRT in spatial WM performance, which were found in experiment 1. However, there were no significant stimulation effects on digit 3-back task. Overall, the findings suggest that offline taVNS has potential on modulating WM performance.

## Introduction

Working memory (WM), a core component of higher cognitive functions, is a system that combines attentional control with temporary storage and information manipulation (Chiesa et al., 2011). The field of cognitive psychology has underlined the importance of the ability to maintain and manipulate information over a period of seconds in WM and the vital role of WM for complex mental abilities, including problem solving, reasoning, and learning (Baddeley and Hitch, 1974). Specifically, one of the central limitations of human cognition is the restricted amount of information that can be kept in WM (Cowan, 2001), and the differences in WM capacity among individuals are associated with variation in several important abilities such as academic performance (Gathercole et al., 2003), non-verbal reasoning ability (Kyllonen and Christal, 1990), and control of attention (Kane et al., 2007). Many clinical populations, including individuals with schizophrenia, attention-deficit/hyperactivity disorder (ADHD), stroke, and traumatic brain injury, also exhibit a lower WM capacity. Moreover, deficits in WM play a crucial role in normal neurocognitive aging and the rapid cognitive deterioration associated with dementias, such as Alzheimer’s disease (Park and Reuter-Lorenz, 2009; Grady, 2012). Fortunately, researches at the beginning of the 2000s showed that the WM was an ability that could be increased by training or psychosocial inventions rather than an immutable individual characteristic (for review, see Constantinidis and Klingberg, 2016). Therefore, the available ways to improve the capacity of WM are urgently needed.

At present, non-invasive brain stimulation techniques, such as transcranial direct current stimulation (tDCS; Andrews et al., 2011; Arkan, 2019; Živanović et al., 2021), transcranial alternating current stimulation (tACS; Ermolova et al., 2019; Benussi et al., 2021), and transcranial magnetic stimulation (TMS; Chen et al., 2015; Hulst et al., 2017), have become one of the mainstream clinical treatment approaches to moderate the WM because of their potential, convenience, and safety. Although some of previous studies have demonstrated the availability of transcranial current stimulation on modulating WM by altering the activity of neurons through changing the resting membrane potential of neurons (Bindman et al., 1962; Nitsche and Paulus, 2000), some recent studies found limited positive effects of tDCS on WM accuracy with a minor reaction time enhancement in healthy cohorts (e.g., Koshy et al., 2020; Shires et al., 2020; for meta-analysis, see Hill et al., 2016; Medina and Cason, 2017). Indeed, the effect of tDCS on WM heavily relies on the stimulation form (online/offline), stimulation duration, current density, and stimulation area (right or left dorsolateral/ventral lateral prefrontal cortex, posterior parietal cortex, or premotor cortex, etc.), which have been further studied (e.g., Nikolin et al., 2018; Živanović et al., 2021). The same problems also appeared in tACS and TMS studies (Chung et al., 2018; Pavlov et al., 2020). At the same time, transcutaneous auricular vagus nerve stimulation (taVNS), as an emerging cranial nerve stimulation method, represents a promising alternative (van Leusden et al., 2015).

The cranial nerves are a specialized part of the peripheral nervous system that emerges directly from the brain rather than through the spine. For each cranial nerve, there is a relatively accessible portion, and each of them is intimately linked to perception and regulation of central nervous system, with “bottom-up” functions in cognition and clinical disorders, which makes them a special target for neuromodulation. The vagus nerve, which is made up of approximately 80% sensory afferent fibers, is the longest cranial nerve (Agostoni et al., 1957). It projects to the nucleus tractus solitarii (NTS) in the medulla, before it is relayed further to other brainstem nuclei and higher-order structures, including the thalamus, hippocampus, amygdala, and insula (Goehler et al., 2000; Saper, 2002). Since the end of the last century, multiple studies in clinical populations have found the special enhancement of vagus nerve stimulation (VNS) on cognition and memory (e.g., Clark et al., 1999; Schachter, 2004; Ghacibeh et al., 2006; Merrill et al., 2006). Recently, some brain imaging studies found that, taVNS, a non-invasive neurostimulation technique that targets the auricular branch of the vagus nerve, produced increased blood oxygen level–dependent signal in the contralateral postcentral gyrus, bilateral insula, frontal cortex, right operculum, and left cerebellum (Badran et al., 2017). Yakunina et al. (2017) suggested that taVNS could modulate the activities in the locus coeruleus (LC) and the areas innervated by this region, including the insula, hippocampus, amygdala, and thalamus. As frontal cortex, hippocampus, and the neurotransmitters, such as norepinephrine (NE), which is released by LC, are known to be important for many cognitive functions, including WM (Gu, 2002; Duffau, 2006; Funahashi, 2017), taVNS gains ever-increasing scientific interest in cognition modulation. In healthy volunteers, several clinical studies have demonstrated that taVNS could modulate a series of cognitive processes, such as emotion recognition (Colzato et al., 2017), divergent thinking (Colzato et al., 2018), inhibitory control processes (Beste et al., 2016), response selection functions (Steenbergen et al., 2015), conflict adaptation (Fischer et al., 2018), attentional processes (Ventura-Bort et al., 2018), and post-error slowing (Sellaro et al., 2015), and so on. In addition, after the first study to explore the effect of taVNS on memory performance (Jacobs et al., 2015), studies have investigated the enhancement of taVNS on verbal memory (Mertens et al., 2020), high-confidence recognition memory (Giraudier et al., 2020), memory reinforcement (Hansen, 2019), long-term emotional episodic memory (Ventura-Bort et al., 2021), and associative memory (Jacobs et al., 2015).

Nevertheless, most of these studies did not refer to WM performance; thus, the effect of taVNS on WM is still unknown. Although direct evidence has been scant, the findings from invasive VNS have shown that the VNS over the left cervical vagus nerve improved immediate WM of epilepsy patients (Sun et al., 2017). Therefore, it is valuable to explore the immediate regulatory effect of taVNS on WM. In the current study, we aimed to investigate the effects of taVNS on WM in healthy volunteers by using n-back tasks. There were two specific questions: first, is there any difference between online and offline taVNS protocols in modulation effect on WM? Up to now, both online (e.g., Jacobs et al., 2015; Colzato et al., 2018; Giraudier et al., 2020) and offline (e.g., Alicart et al., 2020; Warren et al., 2020) taVNS could be seen in researches, and to the best of our knowledge, none of the studies have compared their efficiency. Meta-analyses of tDCS studies have suggested that for healthy population the significant effect could be found only in offline stimulation (see Hill et al., 2016), which might be caused by different neurobiological processes; namely, the online effects might result from resting membrane potential alterations, whereas the offline effects appear to result from modulation of synaptic plasticity (Stagg and Nitsche, 2011; Medeiros et al., 2012; Hill et al., 2016). In addition, the neurotransmitter release needs time to take effect, which might lead to a stronger effect of offline protocol than online stimulation. However, several studies, such as that of Neuser et al. (2020), reported a significant online taVNS effect on motivation. Thus, we compared the effects of online and offline taVNS in the first experiment. Second, are there some generalization effects of taVNS on modulating WM performance, namely, does taVNS have effects on more than one modality of WM tasks? As taVNS has extensive activation on cerebral cortex (Yakunina et al., 2017) and the neurotransmitters released by taVNS might affect a series of cognition, it might have a comprehensive effect on WM performances. However, according to previous studies, there were different neural bases for verbal (like digit) and non-verbal (like spatial) WM tasks (Owen et al., 2005). Thus, the specific effects of taVNS on different modalities and WM tasks are valuable to investigate. To testify this question, we used spatial WM tasks in the first experiment because it has been heavily investigated, and numerous studies have found that it could be improved by the increased activity of prefrontal neurons and dopaminergic transmission (for review, see Constantinidis and Klingberg, 2016) and then tested the corresponding taVNS effects on digit WM tasks in the second experiment. To sum up, in this study, we aimed at (1) investigating the taVNS effects on spatial WM performance and choosing the optimal stimulation protocol between online and offline stimulation and (2) replicating the taVNS effects on spatial WM performance and further investigating its influence on digit WM tasks.

## Materials and Methods

### Experiment 1

In this experiment, we aimed to investigate the enhancement of taVNS on spatial WM by using online taVNS, offline taVNS, and sham groups with two n-back tasks, namely 1-back and 3-back tasks. The behavioral changes between baseline and posttest per condition (online taVNS, offline taVNS and sham) were calculated to evaluate the effects of taVNS on WM.

#### Participant

Forty-eight healthy students at Xidian University were included in this experiment. Each of them had to participate in three sessions, including online taVNS, offline taVNS, and sham. All participants were right-handed, with no smoking, neurological disease, or brain damage history. No participants reported ear injuries, drinking, or taking drugs 48 h before the experiment. Before the experiment, participants were provided with information about the stimulation procedure and experimental protocols and written informed consent. Participants were instructed that they could withdraw from the experiment at any time if they did not wish to continue, and all of them could receive corresponding remuneration. All research procedures were conducted in accordance with the Declaration of Helsinki and approved by the institutional research ethics committee of Xijing Hospital of the Air Force Medical University (KY20192008-X-1). Finally, 46 participants completed the experiment successfully (25 female, average age = 20.39 ± 1.96 years, range = 18–25 years), whereas two subjects were excluded from the data analysis because of withdrawing.

#### Design

**FIGURE 1:**
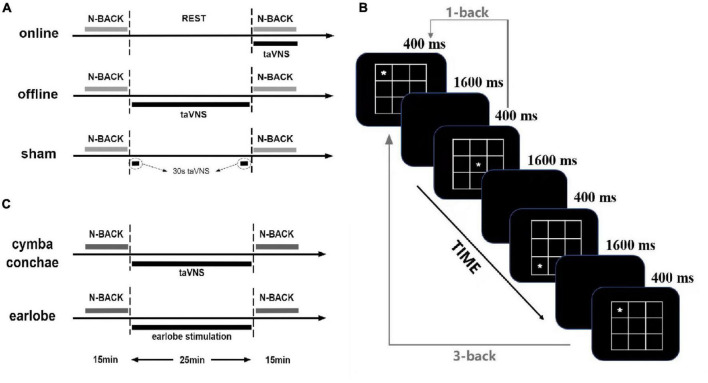
Overview of the study and n-back tasks: **(A)** the protocol of experiment 1; **(B)** the paradigm of spatial n-back tasks, changing the spatial stimuli by digits is the digit 3-back task; **(C)** the protocol of experiment 2.

The experiment was a within-subjects design, with each participant completing three separate sessions, which were different in stimulation conditions [i.e., online taVNS, offline taVNS, and sham (stimulation equipment placebo); Figure 1A]. In the online taVNS condition, participants first tested the baseline of behavioral tasks and then had a 25-min rest. Then, they received a taVNS stimulation at the beginning of the posttest of behavioral task until the end, which lasted approximately 15 min. In the offline taVNS section, participants completed 15-min baseline test of the behavioral tasks, a 25-min taVNS stimulation, and a 15-min posttest of behavioral tasks in turn. The process of sham condition was similar with offline taVNS, except that the 25-min stimulation was instead by a 30-s stimulation at the beginning and end time. The three sessions were separated by a period of at least 2 days (*M*_days_ = 3.46 ± 1.50), and the stimulation orders were counterbalanced between participants. One or 2 days before the formal experiment sessions, participants needed to come to the laboratory to familiarize themselves with the experimental procedure, practice the behavioral tasks (completed whole tasks until reached an accuracy rate of 60%), and test the acceptability of taVNS.

#### taVNS Stimulation Equipment and Parameters

The electrical stimulation equipment used in this study was made by our joint laboratory (XD-Kerfun BS-VNS-001), an upgrade version of the one that has been successfully used in previous researches (e.g., Shen et al., 2021; Sun et al., 2021). The taVNS channel was connected to two silver chloride electrodes (outer diameter 7 mm). The anode and cathode of taVNS were both placed in the left cymba conchae with the cathode inside and 0.5 cm apart from the anode. The electrical stimulation waveform was a single-phase rectangular pulse with a pulse width of 500 μs and frequency of 25 Hz. The current was delivered with a cycle of 30 s on and 30 s off to avoid habituation.

As perceived and tolerated stimulation intensity varies across participants, the current intensity was determined by each participant by using the threshold method to match the subjective experience of the stimulation. Before formal test of each session, there was a threshold test. In the threshold test section, participants were asked to give direct feedback on their feeling of each stimulation intensity on a 10-point scale ranging from (1) no perception to (3) light tingling to (6) strong tingling to (10) intense pain. The stimulation started with an intensity of 0.1 mA and increased stepwise in 0.1-mA increments until the subject reported a slight feeling of pain (corresponding to ≥7 on the subjective sensation scale) and then decreased in 0.1-mA increments until 0.1 mA below the light tingling threshold (corresponding to ≤3 on the subjective sensation scale). The protocol was repeated twice, and the average of the intensities rated as 5 (mild tingling) was used as the stimulation threshold (Neuser et al., 2020). The individual stimulation intensities varied from 0.1 to 1.3 mA for the online taVNS group (*M*_online_ taVNS = 0.7 ± 0.36 mA) and from 0.2 to 1.6 mA for the offline taVNS group (*M*_offline_ taVNS = 0.69 ± 0.38 mA). For the sham group, all the participants tested only the threshold that varied from 0.3 to 1.5 mA and received an intensity at the beginning and the end of the stimulation section for 30 s (*M*_sham_ = 0.73 ± 0.27 mA).

#### Working Memory Tasks

The n-back task is one of the most frequently used paradigms in the assessment of WM capacity, which needs continuous updating of the transient memory storage with novel stimuli in order to compare the new stimuli with previously presented ones (Jarrold and Towse, 2006). In this experiment, we used 1- and 3-back tasks with spatial stimuli. There were four blocks (1-back, 3-back, 1-back, 3-back) with 72 experiment trials in each block. Each block was separated by a 30-s rest period. Before these four blocks, there was a training block with 16 trials for 1-back and 3-back tasks, respectively. Participants were instructed to press “F” when the site of the symbol (“*”) was the same as in one or three trials earlier (namely, “matching” trial), but otherwise pressed “J” (namely, “mismatching” trial, Figure 1B for detailed parameters). Each trial was inserted as picture format with 257 × 257 pixels of width and height. One-third of the trials were matching. Training trials were before experiment trials, and participants could not start the formal experiment unless their training accuracy rate reached more than 75% and the average reaction time was less than 1,000 ms. Psychology experiment computer program E-Prime version 3.0 was used to administer the tasks and record response accuracy and reaction time of all the participants.

### Experiment 2

Based on the design in experiment 1, in experiment 2 we used an active sham group (stimulation placebo), that is, earlobe stimulation group, which was used widely in taVNS modulation studies (e.g., Giraudier et al., 2020; Mertens et al., 2020; Neuser et al., 2020) to further replicate the results that were found in experiment 1. Besides, we added a 3-back task of digit to explore the generalization effect of taVNS on different WM tasks.

#### Participants

Sixty healthy students at Xidian University were included. The inclusion criteria were the same as in experiment 1, mainly including the right handedness, no smoking, no neurological disease, and no brain damage history. No participants reported ear injuries, drinking, or taking drugs 48 h before the experiment. One subject was excluded because of confusing matching and mismatching response, and another subject was excluded because of low baseline accuracy (<30%). Finally, there were 58 students in data analysis (24 female students; average age = 19.90 ± 1.49 years, range = 18–23 years). Each participant was provided written informed consent, and all research procedures were conducted in accordance with the Declaration of Helsinki and approved by the institutional research ethics committee of Xijing Hospital of the Air Force Medical University (KY20192008-X-1).

#### Design

The experiment was a within-subjects design, too, with each participant completing two separate sessions, which were different in stimulation conditions [i.e., offline taVNS and offline earlobe stimulation (offline ES); Figure 1C]. Despite the stimulation site, all the other conditions were the same in the two groups. There was at least a 2-day period (*M*_days_ = 2.93 ± 0.49) between two sessions.

#### taVNS Stimulation Equipment and Parameters

All the information of taVNS and stimulation intensity threshold was the same as in experiment 1, except that the anode and cathode of taVNS were both placed in the left earlobe for the active sham group with anode front side and cathode back side. The stimulation intensity threshold was tested in the same way as in experiment 1, with stimulation intensities varying from 0.3 to 2.7 mA for the taVNS group (*M*_off_line taVNS = 0.74 mA ± 0.37) and from 0.3 to 2.4 mA for the earlobe group (*M*_earlobe–sham_ = 0.84 mA ± 0.39). Both offline taVNS and offline ES groups would receive a 25-min stimulation between baseline test and posttest.

#### Working Memory Tasks

Both spatial and digit 3-back tasks were used in this experiment with two blocks of each form. In total, there were four blocks (spatial, digit, spatial, and digit) with 72 trials in each block. The spatial 3-back paradigm was the same as in experiment 1. The procedure of digit 3-back task was the same as in spatial 3-back task, but the stimuli were changed from the site of “*” to nine Arabic numbers (1, 2, 3, 4, 5, 6, 7, 8, 9). The font of each number was Times New Roman, and the font size was 72. Participants were instructed to press “F” when the number was the same as in three trials earlier, but otherwise pressed “J.” One-third of the trials were matching, and there was a 30-s period between each block. The paradigms and requirement of training blocks were the same as in experiment 1.

### Data Analysis

There are four indicators that are often used, that is, hits (the accuracy of matching trials), correct rejections (the accuracy of mismatching trials) or false alarms (one minus correct rejections), *d* prime (*d*’, hits minus false alarms), and reaction time (e.g., Jongkees et al., 2017). *d*’ was first introduced based on signal detection theory to avoid distorted hits by false alarms (Haatveit et al., 2010). However, it is more of a receptivity indicator than a WM memory ability indicator, which mainly focused on participants’ reaction tendency (Macmillan and Creelman, 1991). In a task with unbalanced matching and mismatching trials, there might be different change tendencies of hits and false alarms, whereas *d*’ might weaken or even conceal these changes (e.g., Haatveit et al., 2010). As the present study was implemented in a healthy cohort whose improvement potential of WM is small, we used both hits and correct rejections (has similar power with false alarms), rather than *d*’, as indicators to avoid missing any changes. Besides, we used the mean reaction time for accurate trials (aRT). Thus, three indicators were calculated for both experiments in the baseline (T0) and during stimulation/poststimulation (T1) of each condition for each participant. For each experiment, the trial if participant missed to response was regarded as a response error. One-way repeated-measures analysis of variance (ANOVA) and paired *t* test were used to check whether the indicators in T0 matched across conditions. The statistical analyses were performed in SPSSv26 (IBM Corp., Armonk, NY, United States) and MATLAB2019b (The MathWorks, Natick, MA, United States).

In experiment 1, the effect of taVNS on indicators were first assessed by using 2 × 3 repeated-measures ANOVA, with both time (T0, T1) and condition (online taVNS, offline taVNS, and sham) as within-subjects factors. *Post hoc* effect analysis was used in significant interaction effects via the paired *t* test for time (T0 vs. T1). In addition, one-way repeated-measures ANOVAs were used to directly compare the change scores from baseline (Δ score = T1 - T0) between conditions. Bonferroni correction was used to explore any significant effects.

In experiment 2, the effects of taVNS on indicators were first assessed using 2 × 2 repeated-measures ANOVAs, with both time (T0, T1) and condition (offline taVNS, and offline ES) as within-subjects factors. *Post hoc* effect analysis was used in significant interaction effects via the paired *t* test for time (T0 vs. T1). In addition, the paired *t* test was used to directly compare the change scores from baseline (Δ score = T1 - T0) between conditions. Bonferroni correction was used to explore any significant effects.

## Results

### Experiment 1

There was no significant difference in baseline performance among the three conditions. There were no obvious feeling difference and adverse reactions of both online and offline taVNS, compared with the sham group (see Supplementary Material for detailed information).

#### Effects of taVNS on Spatial 1-Back Task

**FIGURE 2:**
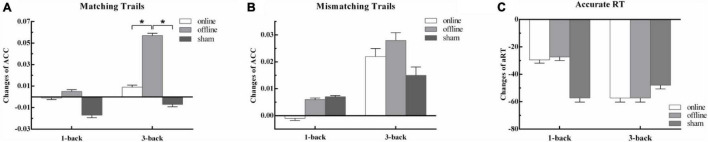
The behavior outcomes of experiment 1. Task outcome changes (T1 - T0): **(A)** the change of accuracy rate for per condition in matching trials; **(B)** the change of accuracy rate for per condition in mismatching trials; **(C)** the change of mean reaction time of correct trials for per condition, and the quantities’ units were ms. One asterisk indicates a corrected *p* value less than 0.05.

There was no significant main effect of conditions for hits [*F*(2,90) = 0.18, *p* = 1.00, η_p_^2^ = 0.004], correct rejections [*F*(2,90) = 0.94, *p* = 1.00, η_p_^2^ = 0.02] and aRT [*F*(2,90) = 0.03, *p* = 1.00, η_p_^2^ = 0.001]. The main effect of time was not significant in hits [*F*(1,45) = 0.31, *p* = 1.00, η_p_^2^ = 0.01] and correct rejections [*F*(1,45) = 2.98, *p* = 0.55, η_p_^2^ = 0.06], but was significant in aRT [*F*(1,45) = 9.93, *p* = 0.02, η_p_^2^ = 0.18]. The aRT at posttest was significantly shorter than that at baseline. The two-way interaction between time and groups was not significant in hits [*F*(2,90) = 0.93, *p* = 1.00, η_p_^2^ = 0.02], correct rejections [*F*(2,90) = 1.04, *p* = 1.00, η_p_^2^ = 0.02], and aRT [*F*(2,90) = 0.01, *p* = 1.00, η_p_^2^ < 0.001]. It suggested that both online and offline taVNS had no significant modulation on 1-back spatial WM (Table 1 and Figure 2).

**TABLE 1:** Accuracy and reaction time for n-back tasks per condition of experiment 1.

	Online group				Offline group				Sham group				Statistical test
	Baseline		Post		Baseline		Post		Baseline		Post		
	Mean	SD	Mean	SD	Mean	SD	Mean	SD	Mean	SD	Mean	SD	*F*(2,90)
***1-Back***													
Hits	0.870	0.012	0.869	0.013	0.866	0.013	0.871	0.013	0.872	0.011	0.855	0.018	0.93
Correct rejections	0.970	0.003	0.969	0.007	0.967	0.005	0.973	0.004	0.971	0.003	0.977	0.003	1.04
Accurate RT (ms)	540.86	17.91	511.39	17.31	538.32	17.76	510.96	14.35	537.88	15.93	507.78	13.39	0.008
***3-Back***													
Hits	0.757	0.021	0.766	0.021	0.751	0.023	0.808	0.018	0.758	0.021	0.751	0.026	5.58*
Correct rejections	0.918	0.021	0.940	0.008	0.930	0.021	0.958	0.007	0.930	0.023	0.945	0.008	1.25
Accurate RT (ms)	665.93	26.03	590.60	19.01	663.13	27.27	605.81	20.05	660.51	22.69	612.58	24.21	0.54

*Hits means accuracy in matching trials; correct rejections means accuracy in mismatching trials; accurate RT means specifically referring to reaction time in all correct trials. F values referred to the two-way interaction. One asterisk indicates a corrected p < 0.05.*

#### Effects of taVNS on Spatial 3-Back Task

There was no significant main effect of conditions for hits [*F*(2,90) = 1.08, *p* = 1.00, η_p_^2^ = 0.02], correct rejections [*F*(2,90) = 3.09, *p* = 0.30, η_p_^2^ = 0.06], and aRT [*F*(2,90) = 0.11, *p* = 1.00, η_p_^2^ = 0.002]. The main effect of time was not significant in hits [*F*(1,45) = 5.39, *p* = 0.15, η_p_^2^ = 0.11] and correct rejections [*F*(1,45) = 0.72, *p* = 1.00, η_p_^2^ = 0.02], but was significant in aRT [*F*(1,45) = 32.46, *p* < 0.001, η_p_^2^ = 0.42]. The reaction at posttest was much faster than that at baseline. The two-way interaction between time and groups was not significant in correct rejections [*F*(2,90) = 1.20, *p* = 1.00, η_p_^2^ = 0.03] and aRT [*F*(2,90) = 0.54, *p* = 1.00, η_p_^2^ = 0.01], but was significant in hits [*F*(2,90) = 5.58, *p* = 0.03, η_p_^2^ = 0.11]. The detailed information is presented in Table 1. *Post hoc* analysis showed that there were no differences between baseline and posttests in online taVNS [*t*(45) = 0.07, *p* = 1.00] and sham [*t*(45) = -0.45, *p* = 1.00] groups, whereas there was a significant improvement in the offline aVNS group [*t*(45) = 4.04, *p* = 0.001]. One-way repeated-measures ANOVA of the Δ score (T1 - T0) among the online taVNS, offline taVNS, and sham groups found a significant difference [*F*(2,90) = 5.59, *p* = 0.005], which showed that the Δ score of the offline taVNS group was significantly higher than that of the online group (*d* = 0.05, *p* = 0.02) and sham group (*d* = 0.06, *p* = 0.01). The results are shown in Figure 2.

### Experiment 2

There was no significant difference in baseline performance between the two conditions. There were no obvious feeling difference and adverse reactions of both offline taVNS and offline ES (see Supplementary Material for detailed information).

#### Effects of taVNS on Spatial 3-Back Task

**FIGURE 3:**
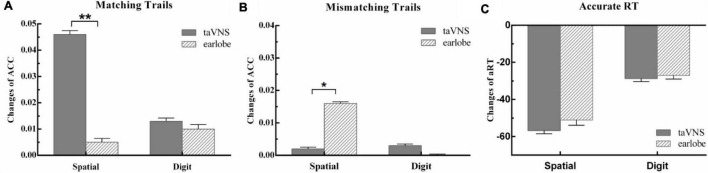
The behavior outcomes of experiment 2. Task outcome changes (T1 - T0): **(A)** the change of accuracy rate for per condition in matching trials; **(B)** the change of accuracy rate for per condition in mismatching trials; **(C)** the change of mean reaction time of correct trials for per condition, and the quantities’ units were ms. One asterisk indicates a corrected *p* value less than 0.05, and two asterisks indicate a corrected *p* value less than 0.01.

The main effect of stimulus site for hits [*F*(1,57) = 3.78, *p* = 0.34, η_p_^2^ = 0.06], correct rejections [*F*(1,57) = 0.81, *p* = 1.00, η_p_^2^ = 0.01], and aRT [*F*(1,57) = 0.42, *p* = 1.00, η_p_^2^ = 0.01] was not significant. The main effect of time was significant in aRT [*F*(1,57) = 15.87, *p* = 0.001, η_p_^2^ = 0.22] and was marginal significant in hits [*F*(1,57) = 7.36, *p* = 0.05, η_p_^2^ = 0.11] and correct rejections [*F*(1,57) = 6.73, *p* = 0.07, η_p_^2^ = 0.11]. The aRT of posttest was shorter than that at baseline, and the hits at posttest were higher than those at baseline, whereas the hits and correct rejections at posttest were higher than those at baseline. The two-way interaction between time and groups was not significant in aRT [*F*(1,57) = 0.07, *p* = 1.00, η_p_^2^ = 0.001], but was significant in hits [*F*(1,57) = 11.32, *p* = 0.006, η_p_^2^ = 0.17] and correct rejections [*F*(1,57) = 9.36, *p* = 0.02, η_p_^2^ = 0.14]. The detailed information was presented in Table 2. *Post hoc* analysis of hits showed that there were no differences between baseline and posttests in earlobe group [*t*(57) = 0.41, *p* = 1.00], whereas there was a significant improvement in the taVNS group [*t*(57) = 4.25, *p* < 0.001]. The paired *t* test of the Δ score of hits between the two groups showed a significant difference [*t*(57) = 3.36, *p* = 0.001], which suggested that the Δ score of the taVNS group was significantly higher than that of the earlobe group (Figure 3). The paired *t* test of the Δ score of correct rejection between the two groups showed a significant difference [*t*(57) = −2.22, *p* = 0.03], which suggested that the Δ score of the taVNS group was significantly lower than that of the earlobe group (Figure 3).

**TABLE 2:** Accuracy and reaction time for spatial and digit 3-back tasks per condition of experiment 2.

	taVNS group				Earlobe group				Statistical test
	Baseline		Post		Baseline		Post		
	Mean	SD	Mean	SD	Mean	SD	Mean	SD	*F*(1,57)
***Spatial 3-back***									
Hits	0.825	0.018	0.870	0.014	0.828	0.021	0.832	0.017	11.32**
Correct rejections	0.965	0.005	0.967	0.005	0.955	0.006	0.970	0.004	9.36*
Accurate RT (ms)	612.22	21.92	555.42	19.81	618.12	25.61	567.06	22.07	0.07
***Digit 3-back***									
Hits	0.871	0.015	0.885	0.013	0.868	0.020	0.878	0.016	0.04
Correct rejections	0.972	0.004	0.975	0.005	0.976	0.003	0.976	0.003	0.32
Accurate RT (ms)	525.66	19.42	496.91	17.81	533.56	19.98	506.53	17.09	0.01

*Hits, correct rejections, accurate RT, and F values have the same meaning with Table 1. One asterisk indicates a corrected p < 0.05, and two asterisks indicate a corrected p < 0.01.*

#### Effects of taVNS on Digit 3-Back Task

There was no significant main effect of conditions for hits [*F*(1,57) = 0.25, *p* = 1.00, η_p_^2^ = 0.004], correct rejections [*F*(1,57) = 0.50, *p* = 1.00, η_p_^2^ = 0.01], and aRT [*F*(1,57) = 0.63, *p* = 1.00, η_p_^2^ = 0.01]. The main effect of time was not significant in hits [*F*(1,57) = 1.76, *p* = 1.00, η_p_^2^ = 0.03] and correct rejections [*F*(1,57) = 0.55, *p* = 1.00, η_p_^2^ = 0.01], but was significant in aRT [*F*(1,57) = 7.99, *p* = 0.04, η_p_^2^ = 0.12]. The aRT at posttest was significantly shorter than that at baseline. The two-way interaction between time and groups was not significant in hits [*F*(1,57) = 0.04, *p* = 1.00, η_p_^2^ = 0.001], correct rejections [*F*(1,57) = 0.32, *p* = 1.00, η_p_^2^ = 0.01], and aRT [*F*(1,57) = 0.01, *p* = 1.00, η_p_^2^ < 0.001]. It suggested that both taVNS and earlobe groups had no significant modulation on 3-back digit WM (Table 2 and Figure 3).

## Discussion

The current study assessed the effects of taVNS on WM performance under varying conditions: online and offline protocols, stimulation equipment sham and active sham (earlobe stimulation), 1-back and 3-back spatial WM tasks, and spatial and digit modalities of WM tasks. Overall, the experiments yielded relatively robust findings about the improvement of taVNS on offline spatial WM performance, no matter compared with online protocol, equipment sham, or active sham group. However, the enhancement of taVNS specifically appeared in offline 3-back spatial WM tasks, but not in online 1-back spatial or 3-back digit tasks.

To the best of our knowledge, this is the first study to investigate the immediate enhancement of WM by taVNS in healthy adults. In the first experiment, we discovered the improvement of offline taVNS on spatial WM capacity by comparing with online taVNS and sham groups, whereas in the second experiment, we replicated the results in experiment 1 with an active sham (offline ES) group. With the exploration and replication samples, we put relatively robust results about the improvement of offline taVNS on WM. There might be three reasons for the improvement. First, as we know, the vagus nerves project to the NTS in the medulla, before being relayed further to other brainstem nuclei and higher-order structures, including the thalamus, hippocampus, amygdala, and insula (Goehler et al., 2000; Saper, 2002). When the vagus nerve projects to the NTS and activates the noradrenergic neurons in the LC and cholinergic neurons in the nucleus basalis, NE and acetylcholine consequently release in wide areas of the cortex (Gu, 2002; Hassert et al., 2004; Roosevelt et al., 2006; Nichols et al., 2011). Subsequently, α1-adrenergic receptors in the dorsal raphe nucleus are activated by NE and release serotonin (Manta et al., 2009). These neurotransmitters can enhance behavioral and cognitive processes, including WM capacity by facilitating neural plasticity (Gu, 2002; Duffau, 2006). Second, long-term potentiation (LTP) as a process involving persistent strengthening of synapses that leads to a long-lasting increase in signal transmission between neurons is widely recognized as a cellular mechanism of memory formation (Bliss and Collingridge, 1993; Bear and Malenka, 1994). As NE is known to facilitate this early LTP through activating β-noradrenergic receptors, the VNS-induced LC-NE release system has been proposed as another possible mechanism of modulating memory performance (Harley, 2007; Mueller et al., 2008). Third, attentional mechanisms might contribute to the improvement of taVNS on WM performance. WM and attention are interacting constructs and tightly intertwined, as attention provides the basis for selecting what information will be encoded in WM (Awh et al., 2006). Previous studies found that VNS could increase early visual N1 amplitude, which is similar to what is seen with increased level of attention (Mangun and Hillyard, 1991; Luck and Ford, 1998). Sun et al. (2017) further discovered that VNS could increase the WM capacity of epilepsy patients by attentional mechanisms.

However, the improved effect of taVNS on WM was absent in the online stimulation group. One would argue that the absence of enhancement in online condition can be attributed to the shorter stimulation time (approximately 15 min) compared with the offline group (25 min). This is possible, but not highly plausible, as no studies have found compelling evidence that increasing stimulation time led to stronger effects on cognitive performance. In fact, the stimulation time of online taVNS in previous studies was highly dependent on the length of behavioral tasks, which varied from 13 to 75 min (e.g., Giraudier et al., 2020; Neuser et al., 2020; Tona et al., 2020), whereas the positive results did not increase with the increase in stimulation time. Besides, if the timing was crucial, there is probably some systemic difference between the 1- and 3-back tasks, which are completed in turn, but this was not the case. Further researches are needed to investigate the specific effects of stimulation time. However, the most likely explanation in the current studies lies in different mechanism behind online and offline taVNS effects, which need more researches. However, as discussed previously, the effect of taVNS on WM mainly depends on the LC-NE release system, which need time to take effect, and this might be the first possible explanation. Furthermore, researches from tDCS showed that there was a trend toward improvement for offline WM performance but was not on online performance in the healthy subjects, whereas the neuropsychiatric cohort exhibited an opposite pattern (e.g., Živanović et al., 2021; for review, see Hill et al., 2016). As online tDCS alters neuronal firing by changing membrane potential, whereas the aftereffects of tDCS stem from changes in synaptic strength, these authors attributed their findings to the optimal cortical excitation/inhibition balance and insufficient neuronal excitability changes in online stimulation in healthy adults. The same pattern appeared here, as Sun et al. (2017) found an online VNS effect in epilepsy patients, whereas this study showed an offline taVNS effect in healthy participants. Thus, we consider that there might be a similar reason that the insufficient vagus nerve excitability changes during online taVNS in healthy adults restrict behavioral changes. Finally, the distracting effect of online stimulation might cover up the modulation effect of online taVNS, and this should be taken into account in further studies.

Beyond the stimulation protocols, the modulation of taVNS might also depend on the properties of the output measure used. Namely, although n-back is a typical paradigm for WM assessment, the numbers of steps back, for example, 1-back (Sandrini et al., 2012), 2-back (Keshvari et al., 2013; Hill et al., 2019), and 3-back (Hill et al., 2019; Živanović et al., 2021), as well as the stimuli of the task, for example, spatial (Živanović et al., 2021), letters (Hill et al., 2019), digits (Nozari and Thompson-Schill, 2013), and objects (Keshvari et al., 2013), are highly variable in the literature. The current study specifically found the taVNS effect on 3-back spatial WM task but not on spatial 1-back or digit 3-back tasks. A meta-analysis suggested that the verbal n-back, like the digit n-back task, was associated with enhanced activation in the left ventrolateral prefrontal cortex, whereas the non-verbal location n-back task was associated with enhanced activation in a set of regions that have been described as a spatial attention network, including right dorsolateral prefrontal, lateral premotor, and posterior parietal cortex (Owen et al., 2005). Given the discussion above, we know that the attentional mechanisms might be one of the reasons for the taVNS effect on WM performance. The improved selective attention induced by VNS (Sun et al., 2017) might have a larger effect on the spatial attention network and contributed to the difference in the improvement of taVNS on spatial and digit WM performance. Besides, the researches in tDCS found that the modulation of electric field on WM depends on the baseline performance (e.g., Assecondi et al., 2021). The individuals or tasks with lower baseline outcome were more likely to have a higher improvement. For 1-back spatial task, the high baseline performance might restrict the modulation of taVNS. It should be noted that the baseline performance of the digit 3-back task was similar to that of the spatial 1-back task, and it might be another probable reason for the uselessness of taVNS. Unfortunately, the present study did not use a digit WM with higher difficulty, and further researches are needed.

Finally, except the difference between the effective stimulation protocol by Sun et al. (2017; online stimulation) and the current study (offline stimulation), these two studies found that for both epilepsy patients and healthy adults, the increase in WM outcome appeared only in hit reactions but not in reaction time, missing response, or correct rejections. These results show consistency between the clinical study and laboratory investigation, which make these results more convincing. According to Sun et al. (2017), the improvement of selective attention increased accuracy in target trials, that is, hits, whereas the unchanged general level of attention was attributed to the unchanged aRT and correct rejection rate. Besides, the high baseline of correct rejections might restrict the increased potential of posttest, and the improved familiarity of the tasks leads to a comprehensive main effect of aRT in all groups. However, as shown in the meta-analysis, tDCS could improve the reaction time in healthy adults (Hill et al., 2016); the synergistic effects of tDCS and taVNS might lead to a more comprehensive improvement in WM, which has been proven in neuroimaging study (Sun et al., 2021), whereas the effects on behavior need to be investigated in the future. Another interesting finding that should be noted is that the offline ES increased participants’ correct rejection rate. It might be caused by the special effect of nervus auricularis magnus, which was activated by earlobe stimulation. The further effect and mechanism of earlobe stimulation need more studies.

Nevertheless, there are still some limitations to the current study. First, the optimal condition offline taVNS, especially the stimulation time, was not clear in the present study. Although the stimulation time in this study has led to a strong effect, we know little about the effects in longer or shorter stimulation conditions. As the stimulation time influences the convenience and safety and is important for standard protocol, it needs more investigation. Besides, the modulation effect of online taVNS might also depend on stimulation time more or less. The optimal stimulation time might lead to a stronger and more efficient online taVNS effect on behavior performance, which needs more researches in the future. Second, if the taVNS modulation effects exist only in the offline protocol, it is valuable to investigate the potential mechanism difference between online and offline taVNS, such as the excitability changes of vagus nerve, which might put new perspective about the effects of taVNS and need further researches. Third, as mentioned previously, although we failed to identify the generalization effects of taVNS from spatial WM to digit WM task, the absent improvement of taVNS on digit WM performance is not clear in detailed reasons, namely, whether it is caused by the specificity of taVNS or the high baseline performance. Thus, further studies with more difficult digit/verbal WM tasks or some subjects with lower WM capacity, such as aging people or patients, are needed. Fourth, beyond n-back task, there are many other tasks that require WM capacity, like Sternberg task. Therefore, studies with other tasks are needed to further verify the generalization effects of taVNS on WM ability. Lastly, although the immediate improvement of WM was strong after offline taVNS, it is unknown to date whether acute improvements can predict the sustained therapeutic effects of potential taVNS-based treatment. Translation to clinical settings remains as a vital question and urgently needs more researches.

## Conclusion

To summarize, although the vagus nerve is known to play a vital role in the regulation of cognition, the immediate modulatory effects of vagal afferent signals on WM in healthy cohort are largely elusive to date. Here, using taVNS, we demonstrate that stimulation of the vagus nerve increases performance of offline spatial WM tasks in healthy populations, whereas the evidence of improvement of taVNS on digit WM tasks was absent and needs further researches. In general, our results shed light on the role of peripheral physiological signals in regulating WM and highlight the potential for non-invasive cranial nerve stimulation techniques to improve a person’s cognition and behavior.

## Data Availability Statement

The raw data supporting the conclusions of this article will be made available by the authors, without undue reservation.

## Ethics Statement

The studies involving human participants were reviewed and approved by the Institutional Research Ethics Committee of the Xijing Hospital of the Air Force Medical University. The patients/participants provided their written informed consent to participate in this study.

## Author Contributions

J-BS, Q-QT, and WQ: conception and study design. J-BS, Q-QT, X-YG, Y-PC, and CW: data collection or acquisition. J-BS, CC, and Q-QT: statistical analysis. J-BS, CC, HY, X-JY, and HD: interpretation of results. J-BS and CC: drafting the manuscript work or revising it critically for important intellectual content. All authors approval of final version to be published and agreement to be accountable for the integrity and accuracy of all aspects of the work.

## Conflict of Interest

The authors declare that the research was conducted in the absence of any commercial or financial relationships that could be construed as a potential conflict of interest.

## Publisher’s Note

All claims expressed in this article are solely those of the authors and do not necessarily represent those of their affiliated organizations, or those of the publisher, the editors and the reviewers. Any product that may be evaluated in this article, or claim that may be made by its manufacturer, is not guaranteed or endorsed by the publisher.
