# Supplementary material for: Transcutaneous Auricular Vagus Nerve Stimulation Improves Spatial Working Memory in Healthy Young Adults
Source: Front Neurosci. 2021 Dec 23;15:790793. doi: 10.3389/fnins.2021.790793 (PMC8733384; doi:10.3389/fnins.2021.790793)
Supplement: Supplementary file 1 [file Data_Sheet_1.docx]

**Transcutaneous auricular vagus nerve stimulation (taVNS) improves spatial working memory in healthy young adults**

Supplementary materials

**Adverse reaction of taVNS stimulation**

To evaluate people’s tolerance on taVNS and the adverse reactions that taVNS might cause, we collected headache, cervicodynia, nausea, face or neck muscle contractions, prickling, burning sensation, general discomfortableness, and other side-effects information by questionnaire. All the symptom was evaluated by a 5-point scale ranging from (1) quite a lot (2) a lot (3) moderate (4) a little (5) no at all. The questionnaire was collected immediately after post-test. We considered one and two scores as an obvious adverse reaction while three, four and five scores were considered as normal reactions.

In Experiment 1, there were 46 subjects were included in the data analysis. There was no significant difference between online-taVNS, offline-taVNS and sham groups. The mean scores of the symptoms were showed in Table S1. The times of adverse reactions that reported were recorded in Table S2.

In Experiment 2, there were 58 subjects were included in the final data analysis. There was no significant difference between offline-taVNS and offline-earlobe sham groups. The mean scores of the symptoms were showed in Table S3. The times of adverse reactions that reported were recorded in Table S4.

Table S1. Mean scores of all symptoms in each condition.

| Symptoms |  | Online-taVNS |  | Offline-taVNS |  | Sham |
| --- | --- | --- | --- | --- | --- | --- |
| *Headache* |  | 4.54±.66 |  | 4.54±.72 |  | 4.52±.78 |
| *Cervicodynia* |  | 4.65±.71 |  | 4.80±.50 |  | 4.72±.66 |
| *Nausea* |  | 4.83±.49 |  | 4.93±.33 |  | 4.85±.51 |
| *Face or neck muscle contractions* |  | 4.72±.62 |  | 4.89±.38 |  | 4.87±.50 |
| *Prickling* |  | 3.72±.72 |  | 3.72±.81 |  | 3.93±.93 |
| *Burning sensation* |  | 4.50±.81 |  | 4.67±.60 |  | 4.57±.78 |
| *General discomfortableness* |  | 4.26±.80 |  | 4.33±.82 |  | 4.39±.88 |
| *Other side-effects* |  | 4.70±.63 |  | 4.76±.52 |  | 4.76±.60 |

| Symptoms |  | Online-taVNS |  | Offline-taVNS |  | Sham |
| --- | --- | --- | --- | --- | --- | --- |
| *Headache* |  | 0 |  | 1 |  | 1 |
| *Cervicodynia* |  | 1 |  | 0 |  | 0 |
| *Nausea* |  | 0 |  | 0 |  | 0 |
| *Face or neck muscle contractions* |  | 0 |  | 0 |  | 0 |
| *Prickling* |  | 2 |  | 3 |  | 3 |
| *Burning sensation* |  | 2 |  | 0 |  | 1 |
| *General discomfortableness* |  | 0 |  | 1 |  | 2 |
| *Other side-effects* |  | 0 |  | 0 |  | 0 |

Table S2. Times of reported adverse reaction on each symptom in each condition.

Table S3. Mean scores of all symptoms in each condition.

| Symptoms |  | Offline-taVNS |  | Offline-ES |
| --- | --- | --- | --- | --- |
| *Headache* |  | 4.67±.82 |  | 4.79±.55 |
| *Cervicodynia* |  | 4.79±.52 |  | 4.81±.54 |
| *Nausea* |  | 4.84±.45 |  | 4.88±.46 |
| *Face or neck muscle contractions* |  | 4.76±.51 |  | 4.81±.44 |
| *Prickling* |  | 4.02±.69 |  | 4.09±.78 |
| *Burning sensation* |  | 4.71±.56 |  | 4.78±. |
| *General discomfortableness* |  | 4.26±.80 |  | 4.26±.80 |
| *Other side-effects* |  | 4.70±.63 |  | 4.70±.63 |

Table S4. Times of reported adverse reaction on each symptom in each condition.

| Symptoms |  | Offline-taVNS |  | Offline-ES |
| --- | --- | --- | --- | --- |
| *Headache* |  | 3 |  | 1 |
| *Cervicodynia* |  | 0 |  | 0 |
| *Nausea* |  | 0 |  | 0 |
| *Face or neck muscle contractions* |  | 0 |  | 0 |
| *Prickling* |  | 0 |  | 1 |
| *Burning sensation* |  | 0 |  | 0 |
| *General discomfortableness* |  | 0 |  | 0 |
| *Other side-effects* |  | 1 |  | 0 |
